# Intervening factors between risk of violence and aggressive behaviours among forensic inpatients: a scoping review

**DOI:** 10.1186/s40359-024-01649-1

**Published:** 2024-03-15

**Authors:** Norhameza Ahmad Badruddin, AbRahman Roseliza-Murni, Mohammad Rahim Kamaluddin, Abdul Rahman Ahmad Badayai, Shalini Munusamy

**Affiliations:** 1 Centre for Research in Psychology and Human Well-Being, Faculty of Social Sciences and Humanities, Universiti Kebangsaan Malaysia, 43600 Bangi, Selangor Malaysia; 2 International Medical University, Federal Territory of Kuala Lumpur, 126, Jln Jalil Perkasa 19, Bukit Jalil, 57000 Kuala Lumpur, Malaysia

**Keywords:** Violence risk, Aggression, Intervening factor, Forensic inpatients

## Abstract

**Background:**

Risk of violence is closely associated with aggression propensity. However, there is a lack of research to explain the mechanisms behind this association, especially among the patients of forensic secure facilities. This review aimed to identify and synthesize the available literature concerning the intervening factors (mediating or moderating factors) in the relationship between the risk of violence and aggressive behavior in forensic secure facilities.

**Methods:**

Two electronic academic databases were searched: Scopus and Web of Science (WoS) using specific keywords as search terms derived from the PCC framework with no specific time limit. The search strategy was developed based on the JBI Manual for Evidence Synthesis and utilised the PRISMA-ScR guidelines. Data on the risk of violence, intervening factors, and aggressive behavior were extracted from the included studies. Further analysis was performed whereby similar data were grouped and synthesised together.

**Results:**

The initial search produced 342 studies. However, only nine studies fulfilled the inclusion criteria. The nine studies included 1,068 adult forensic inpatients from various psychiatric hospitals. Only mediation studies reported significant mechanisms of influence between the risk of violence and aggressive behavior. It is postulated that the human agency factor may be the underlying factor that influences a person’s functioning and the subsequent series of events between the risk of violence and aggression.

**Conclusions:**

In light of the paucity of evidence in this area, a generalised conclusion cannot be established. More studies are warranted to address the gaps before conclusive recommendations can be proposed to the relevant stakeholders.

## Background

Aggressive behaviour is defined as a spectrum of behaviours that can be conceptualised as dynamic interactions between individual agencies, thoughts, emotions, and actions [1–3]. Aggressive behaviours in the inpatient setting also known as institutional aggression or violence [4]. Despite conceptual differences between aggression and violence, research involving forensic inpatients has operationalized both aggression and violence by referencing to the deviant behavior during institutionalization. Violence was classified if the incident occurred with the presence of clear instigator or co-aggressor and if the incident involved harm to staff or others [5]. Jeandarme et al. (2019) [6] includes verbal violence (threatened violence) and physical violence (attempted or actual) as inpatient violence. On the other hand, Tuente et al. (2021) [7] and Huitema et al. (2021) [8], operationalized forensic inpatient aggression as threats and actual verbal or physical behavior towards others, self or property. Despite a thin difference between these two terms, similar method has been applied in assessing both aggression and violence within the forensic facility [8–11]. Along with that, violation of the institutional rules in any form can also be termed as institutional misconduct. Based on the Importation Model and Deprivation Model, institutional aggression is defined as the interplay between individual characteristics, subcultures, and life limitations within an institution that can subsequently result in frictions that may compromise survival in reality [12, 13]. The outcome of a person’s interaction with a restricted environment often produces different templates of aggression.

Institutionally speaking, forensic inpatient aggression is a pertinent issue. Recent studies reported a high prevalence of aggressive incidents in the forensic inpatient setting [14, 15]. In a recent systematic review, at least 20% of forensic inpatients were involved in any type of physical violence at least once during their stay [16]. Similarly, Verstegen et al. (2020) [17] recorded that as high as 60% of forensic inpatients were involved in any form of aggressive behaviours. To date, most of the incidents are focused on severe physical aggression among inpatients [4, 18, 19]. A recent mapping of aggressive behaviours among 120 forensic inpatients with history of reactive aggression over 30 weeks showed that 37.5% of them were involved in moderate range of physical aggression [7]. Similar to physical aggression, verbal aggression and threatening behaviours are equally common among this group of inpatients as reported in many studies. Very often, these behaviours are the precursor to physical aggression [7, 8].

In addition, forensic inpatient aggression can result in larger systemic impacts [20]. For instance, it may perpetuate to violence, especially among aggressors with poor anger management [21]. At the same time, it creates a climate for victimisation [22], reduces positive group climate [23], and increases staff burnout [24]. Furthermore, it can potentially lead to overcrowding due to longer admission required [25], eventually imposing negative management and financial implications [26, 27].

Risk of violence plays a crucial role in reducing aggression incidence and mitigating the systemic impact based on relevant studies of inpatient aggression [28, 29]. The conceptualisation of aggression is closely related to the static or dynamic factors that can be extracted the from assessment of violence risk. Two recent studies identified more than 70 risks factors for aggression among forensic inpatients [30, 31]. Most of these factors are dynamic rather than static in nature and they are often linked temporally to inpatients aggression [4, 7, 32–34].One of the postulated reasons is the modifiable nature of these factors in terms of their relationship with aggression intervention goals [33, 35].

Risk of violence and aggressive behaviour have been rigorously researched upon since the early 1990s. Most of the researchers focused on aggressive behaviours through the assessment of violence risk [36, 37], including the investigation of the predictive ability of the violence risk assessment [38]. In two recent prediction studies, violence risk assessment has limitation to shed light on understanding the underlying mechanisms that lead to aggression [30, 39]. In a way, this highlights the gap between violence risk assessment and daily practical management of aggression. Furthermore, besides the strength or consistency of the association between risk of violence and aggression, very little is known about the intrinsic relationship between these two construct [40]. There are also a few studies that reported a mismatch between the identified risk and the actual management plan [41, 42].

Realistically speaking, the relationship between risk of violence and aggression is complex, thus making risk management a complicated issue. Very often, existing studies on risk of violence assessment did not take into account individual differences in aggression, with outcomes from risk of violence reported as uncertain. The equivocation in describing aggressive behaviour, especially among forensic inpatients, stemming from the variability in risk factors, a wide range of definitions of aggression, challenges in research methods, and more often than not, the risk management itself [42–44]. Penny (2021) [40], succinctly concluded that the association between risk of violence and aggression should be explored as a process of how the identified risk factors materialised into aggression and how the risk reduced with any given effective management through an intervening agent.

The challenges in addressing forensic inpatient aggression fall in the two major concern namely the predictive performance of violence risk assessment [45–47] and inconclusive evidence of successful desistance from aggressive behavior [48, 49]. Aggression theories indicates that aggressive behavior is regarded as an outcome from the ensemble of mechanism formed together between risk factors for violence and other intrinsic and extrinsic factors [3, 50]. We postulate intrinsic and extrinsic factors potentially intervene the relationship between risk of violence and aggression. However, the understanding of forensic inpatient aggression is challenged with various risk factors of aggression, utility of violence risk assessment in forensic services [51], narrow operationalization of aggression definition, diversity in violence risk management [52, 53] and research methodologies [54]. There have been empirical studies applying intervening model such as the mediation and moderation model to deepen the understanding of aggressive behavior. Thus, the motivation of this review is twofold. We aim to identify and map the intervening factors involved in the relationship between risk of violence and aggressive behavior to expend the understanding of aggression phenomena specific to forensic secure facility population and potentially identify research gap within the interest. The existing literature increasingly shows an unvarying understanding between the relationship of risk of violence and aggressive behavior among the forensic inpatients. O’Dowd et al., (2023) [55] concluded that existing literature on violence risk assessment and aggression management has neglected individual intrinsic resources as an influencing factor to aggression. However, little is known about the intervening factors between the connection of risk of violence and aggression within the forensic inpatient. Hence, identifying intervening factor is relevant.

Thus, this scoping review aimed to systematically examine and synthesise the evidence from published studies that revolved around the intervening factors between risk of violence and aggressive behaviour among forensic inpatients. By identifying and mapping the evidence, this work proposed a gap for future research within the context of risk of violence and aggressive behavior.

## Methods

Scoping review are used to review a body of literature and are defined as studies that aim to identify and map the literature on a particular topic and provide an opportunity to identify gaps in the research, key concepts and sources of evidence [56]. This scoping review was conducted to investigate the factors that have been studied as intervening factors in the connection between risk of aggression and aggressive behavior. Additionally, we identified any potential gaps in the literature concerning the relationship between risk of violence and aggressive behavior within the forensic secure facility. The scoping review was conducted following the JBI nine-step process by Peters and colleagues [57, 58] (Table 1). It also utilised the Preferred Reporting Items for Systematic Reviews and Meta-Analyses extension for Scoping Reviews (PRISMA-ScR) guidelines [59]. This scoping review is not a registered protocol. A scoping review was undertaken to systematically map the research included and identifying any existing gaps in knowledge [57, 60].

**Table 1:** *JBI Scoping Review Framework*

Step 1. Defining and aligning the objective/s and question/s
Step 2. Developing and aligning the inclusion criteria with the objective/s and question/s
Step 3. Describing the planned approach to evidence searching, selection, data extraction, and presentation of the evidence
Step 4. Searching for the evidence
Step 5. Selecting the evidence
Step 6. Extracting the evidence
Step 7. Analysis of the evidence
Step 8. Presentation of the results
Step 9. Summarizing the evidence in relation to the purpose of the review, making conclusions and noting any implications of the findings

Adapted from: JBI 2020, Scoping Review [57]

### Defining the objectives and question

This scoping review aimed to identify and explore factors that have been intervening the connection between risk of violence and aggressive behavior within the forensic secure facility inpatients. This question formed the knowledge gap to further exploring risk of violence utility and gathering insights on aggression phenomena.

#### Objectives

i) What intervening factor have been considered?
ii) What type of violence risk was considered in the intervening model?
iii) What measure was used to determine aggressive behavior?

### Developing the inclusion criteria

The PCC (*Participants, Concept and Context*) framework was utilised in the process of developing the research question, objective, inclusion and exclusion criteria and literature search strategy [57]. The eligibility criteria are directly linked to the research question and objective (Table 2).

**Table 2:** *Objectives, Research Questions and Eligibility Criteria*

Objective	Identify intervening factor that have been studied in the relationship between risk of violence and aggressive behavior within forensic secure facility inpatients, how the factor contributes to the relationship and identify the type of violence risk and aggressive behavior considered in the intervening relationship?		
Research question	What intervening factor have been studied in the relationship between risk of violence and aggressive behavior among forensic secure facility inpatients?		
PCC framework	***Population***: Offenders with mental illness receiving treatment as inpatient in secure facility of any security level or inpatients under the forensic psychiatry services provision.	***Concept***: Any intervening factors including mediator or moderator.	***Context***: The relationship between risk of violence and aggressive behavior.
Eligibility criteria	All studies involving forensic inpatients over the age of 18 years during data collection. With any offence record OR/ AND mental illness.	Mediation studies. Moderation studies. Quantitative research design.	Studies that include at least one variable of violence risk and measurement of aggression. Written in English.

### Eligibility criteria

This scoping review included only quantitative studies that investigate the intervening factors in the relationship between risk of violence and aggression among offenders with mental illness admitted as inpatients in secure facilities under forensic psychiatry services.

#### Inclusion criteria

- Participants aged 18 years and older.
- Participants identified as having an offense record and mental illness.
- Outcome measure defined as aggression of any form and severity.
- Study variables included intervening factors (mediators or moderators).
- Quantitative study design Published in the English language.

#### Exclusion criteria

- Participants included juvenile offenders or those 17 years and below.
- Studies from the perspective of staff.
- Outcome measures that did not support study objectives.
- Qualitative design.

### Approached to finding evidence

Our search strategy (including identifying key words). Search techniques included using medical subjects heading (MeSH) and Boolean operators to extract relevant result (Table 3). Only published studies in the English language were included with no time limit. When full-text article were not available or published in the English language, the author make a request through email. Article that were not made available upon request after 1st December 2022 was excluded from the study.

### Searching for the evidence

The evidence search was undertaken in two stages. Stage one involved an initial brief search on Google Scholar to identify article on the topic. The key words and text words contained in the title and abstract of relevant articles used to developed search strategy (Table 3). Stage two involved searching for evidence on selected electronic databases, namely Scopus and Web of Science (WoS) without any time limitations.

**Table 3:** *Search Strategy Using the PCC Framework*

Keyword	Search words (synonyms/ related terms/ variations)
P, Population: (Forensic inpatients)	(“forensic” OR “psychiatric hospital” OR “secur* facility”)
C, Concept: (Intervening factor)	(mediat* OR “moderat*)
C, Context: (Relationship between violence risk and aggression)	(“violent risk” OR “dynamic risk” OR “modifiable risk” OR “static risk”) AND (“aggress*” OR “violen*”)

### Selecting the evidence

**Fig. 1:**
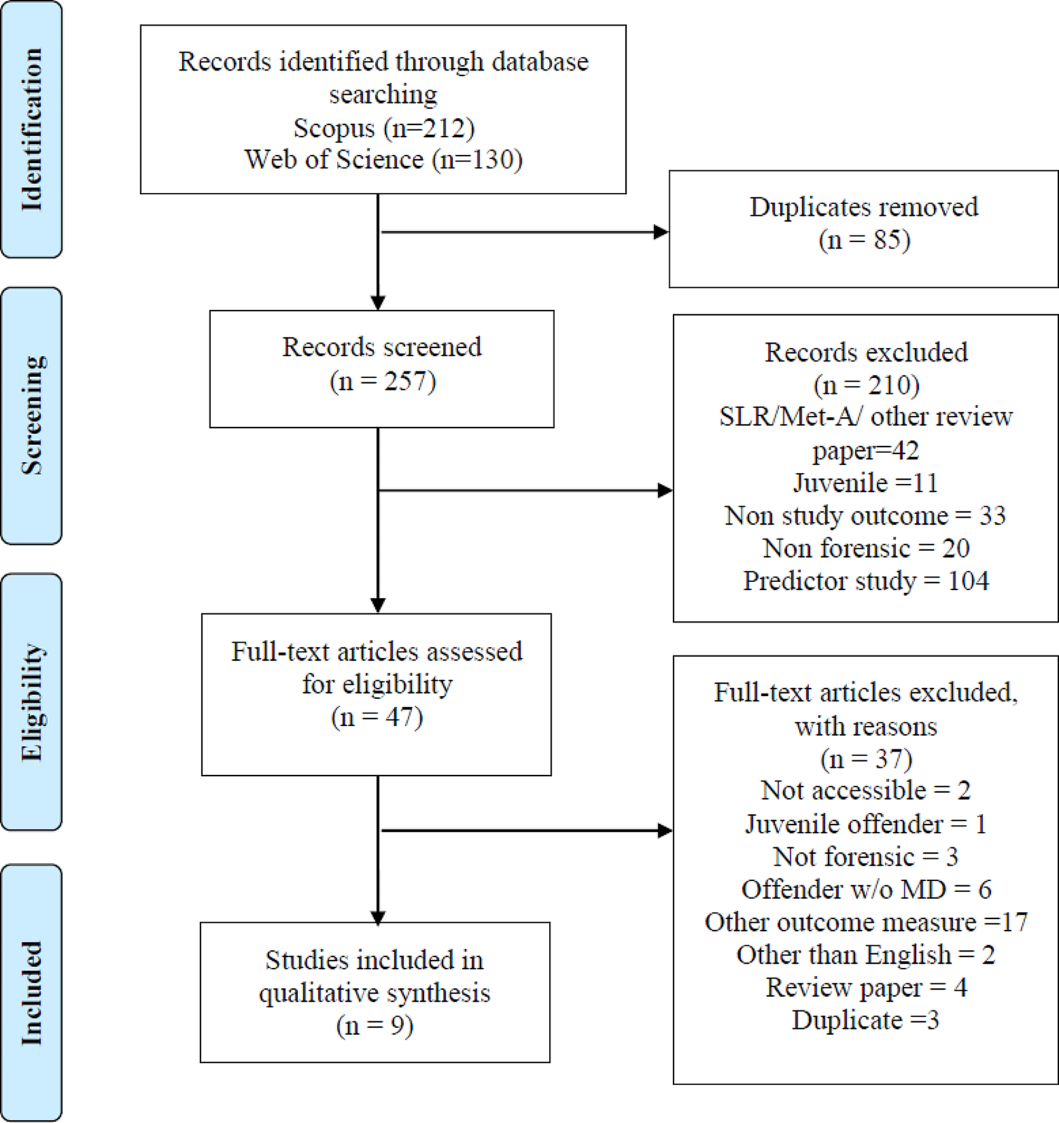
*PRISMA Flowchart of Article Selection Process*

Firstly, relevant studies were retrieved from the databases based on the search string. The retrieved studies were imported into Mendeley Desktop for screening. This was followed by the screening of the titles and abstracts for relevance by the lead author N.A.B. Duplicates, articles that were not relevant, and articles that did not meet the eligibility criteria were removed. After that, full-text papers of studies that fulfilled the eligibility criteria were retrieved for (Table 2). Two independent reviewer (N.A.B, S.M) assessed all the selected studies to ensure that they met the inclusion and exclusion criteria of this review. Any disagreements between two reviewer were managed by consensus and a third author M.R.K was consulted upon disagreement over the suitability to include any articles. The reasons for exclusion at full text are been identified in the PRISMA flowchart (Fig. 1).

### Extracting the evidence

Data extracted from the studies included are article title, authors, journal, publication year, population, concept (intervening variable), context (independent and dependent variables), study design, data analysis, outcome and limitation. Information extracted was analysed whereby similar data were grouped and synthesised together. Study quality were not conducted during the review as the aim of this scoping review was to identify available evidence and knowledge gap [56].

### Analysis of the evidence

The initial search yielded 342 studies from both databases. However, 85 studies were identified as duplicates. The remaining 257 articles were screened based on their titles and abstracts for suitability to be included in this review. Following that, 210 studies were excluded for various reasons. Only 47 full papers were identified as potential studies that reported risk of violence and aggression among forensic inpatients. After the appraisal, another 37 studies were excluded. Nine studies (k = 9) met the selection criteria and were included in this review. The study selection process was conducted following the PRISMA-ScR guideline [59]. A flow chart of the search results is summarised in Fig. 1.

## Results

### Study characteristics

There was a limited number of studies examining intervening factors between risk of violence and aggressive behaviour among forensic inpatients. Only nine studies (k = 9) were deemed as relevant to be included in the final review. Three studies were conducted in the Netherlands [61–63]. Two studies were conducted in the United Kingdom (UK) [5, 64] and in the United States (US) respectively [65, 66]. The other two are from Sweden [67] and Denmark [68] each. The study sites included eight forensic hospitals (k = 8) and one mental hospital (k = 1). Eight of the nine studies were conducted in the past ten years. Only quantitative studies were included in this scoping review of which five were conducted using cross-sectional design [5, 62, 64, 66, 67], three file study [61, 63, 65] and one longitudinal design [68]. Most of studies with a larger sample size used retrospective archival method while the cross-sectional studies in the review included sample sizes ranging from 51 to 98 patients.

A total of 1,068 forensic inpatients with the age of 18-years old and above at the data collection period were included. Male forensic sample (*n* = 953) predominate in almost all studies in which three studies included only male patients [61, 63, 68] while another five had a disproportionately higher number of male patients [5, 62, 64, 65, 67] and one study with unreported gender distribution [66]. Table 4 outlines an overview of the characteristics of the included studies.

**Table 4:** *Characteristics of the Included Studies*

Study	Country Location	n	Gender	Age	Study Design	Analysis
O’Reilly et al., (2015) [5]	Ireland Secure forensic hospital	*n* = 89	84 males 5 females	µ:40	Quantitative; Cross-sectional and prospective observational cohort study	Regression analysis
Garritsen et al., (2022) [61]	Netherlands Forensic hospital	*n* = 315	All male	µ:31.8; SD:8.69 Age range = 17–66	Quantitative; Retrospective file study	Logistic regression
Soe-Agnie et al., (2021) [62]	Netherlands Forensic psychiatry and addiction care hospitals	*n* = 94	78 males 15 females	µ:37.65; SD:9.28	Quantitative; Cross-sectional	Regression analysis
Robbé et al., (2013) [63]	Netherlands Forensic psychiatric hospital	*n* = 188	All male	µ:32; SD:7.3 Age range = 18–56	Quantitative; Retrospective file study	Logistic regression
O’Reilly et al., (2019) [64]	Ireland Central mental hospital	*n* = 55	49 males 6 females	µ:40; SD:9.7	Quantitative; cross-sectional national forensic cohort	Mediation analysis
Green et al., (2016) [65]	New York Forensic hospital	*n* = 124	100 males 24 females	Male µ:46.2; SD:13.2 Female µ:40.9; SD: 11.5	Quantitative; Retrospective file study	Logistic regression
(Welsh & Gordon, (1991) [66]	Philadelphia Federal forensic hospital	*n* = 51	-	(µ:29.1; SD:6.6)	Quantitative; Cross-sectional	Multiple regression
Meddeb et al., (2022) [67]	Sweden Forensic psychiatric clinic	*n* = 98	85 males 13 females	µ:34.9; SD:10.7 Age range 19–62	Quantitative; Cross-sectional	Regression analysis
Moeller et al., (2017) [68]	Denmark Forensic hospital	*n* = 54	All male	µ:36.4; SD:11.9 Age range 19 to 67	Quantitative; Prospective	Binominal regression

### Risk of violence

Risk of violence constitutes multi-faceted risks in the forms of actuarial or Structured Professional Judgement (SPJ). Three of the studies measured the risk of violence using the SPJ approach. Two studies used Historical Clinical Risk-20 (HCR-20) [63, 65] while one applied the Historical Clinical Future-Revised (HKT-R) [61]. Six other studies investigated a single risk factor with an association to aggression [5, 62, 64, 66–68]. Two studies used psychopathology symptoms as a single risk factor [64, 68] while two other studies identified personality traits as risk of violence. Two personality traits that were discussed in the studies were psychopathic trait [62] and hostile personality trait [66]. The remaining two studies used single risk factors such as anti-social behaviour [67]and neuro-cognitive function [5]. Table 5 presents the data obtained from the included studies.

### Aggressive Behaviour

A diverse measure of aggressive behaviours was included as the outcome measures in the studies included (Table 5). Three studies measured aggression incidents that occurred during hospitalisation, ranging from physical aggression [68] to subtle aggressive behaviours such as causing harm to others and aggression towards objects [5, 65]. Additionally, two studies identified aggression outcome as recidivism (i.e., any form of new conviction of any type or form of offence after discharge or unconditional release) [61, 63]. Three studies used self-reported questionnaire in observing aggressive behaviour [62, 64, 67, 69]]. Self-reporting method focuses on externalising behaviours that are reflected in observable aggressive behaviour, irresponsibility, deceitfulness, impulsivity or sensation seeking, and blaming [62, 67]. Apart from that, only one study measure aggression using patients self-report which is the instrumental-reactive aggression [64]. Reactive aggression refers to aggression that is primarily based on emotion, impulsivity, and defensiveness while instrumental aggression is predatory, goal-oriented, cold, and premeditated in nature [64, 69]. Interestingly, one study that was conducted more than a decade ago used aggression observation method via role play assessment [66]. The aggression responses were recorded from an imaginal exposure to provocative stimulus and the aggression responses among the forensic inpatients were videotaped and rated independently by blinded rater. Overall, the included studies applied multiple ways of measuring aggression based on the construct of aggression determined by the researcher, namely observation (incidents or repeated offence and imaginal aggressive responses), as well as patient’s self-report aggressive behaviours.

**Table 5:** *Study Outcomes*

Study (*N* = 9)	Violence risk factor	Method of assessing risk factor	Intervening factor	Outcome measure	Result	Limitation
Moderating factor (*n* = 4)						
Garritsen et al., (2022) [61]	Dynamic factors	HKT-R	Intellectual ability	Recidivism	Intellectual ability had no significant moderating effect on the associations between dynamic risk of violence and violent recidivism.	Gender disproportion Retrospective design
Robbé et al., (2013) [63]	Violence risk	HCR-20 SAPROF	Types of offence	Violent recidivism	None of the offense type are significant moderator in predicting violent recidivism.	Gender disproportion Retrospective design
Green et al., (2016) [65]	Violence risk	HCR-20	Gender	Aggression incidents	Gender was not a significant moderator in predicting likelihood of violence.	Gender disproportion Retrospective design
Meddeb et al., (2022) [67]	Aggressive antisocial behaviour	LHA	Adverse childhood experience	Externalizing behaviour	Adverse childhood experience does not have moderating effect on the association between aggressive antisocial behaviour and disinhibition.	Measurement used was lack of precision Type I error
**Mediating factor (***n* **=** **5)**						
O’Reilly et al., (2015) [5]	Neurocognitive	MCCB	Social cognition	Aggressive incidents	Social cognition has a direct effect on violence independent of neurocognition, violence proneness and symptom severity.	Gender disproportion Small sample size
Soe-Agnie et al., (2021) [62]	Psychopathic traits and Impulsivity	PPI-R BSI-11	Self-deception	Externalizing behaviour	Self-deception does not show mediation effect between impulsivity and externalizing behaviour.	Mediator measure was with low reliability
O’Reilly et al., (2019) [64]	Schizophrenia symptoms	SAPS	Moral cognition	CIRA HCR-20	Moral cognitions mediated the relationship between the presence of specific psychotic symptoms and form of violence.	Cross sectional design can’t conclude causal relation and direction in the mediation model
(Welsh & Gordon, (1991) [66]	Personality Past behaviour Arousal	BDHI STAS SES Type of offence Self-report arousal feelings	Cognitive model TRA	Aggression Role Play Assessment	Cognitive variable partially mediate aggressive behaviour	Small sample size
Moeller et al., (2017) [68]	Psychological distress	Novaco Anger Scale/ HADS/ PSD Check List/ Psychotic symptoms	Violent images	Physical aggression	No mediation effect	Small sample

Note: HKT-R = Historical Clinical Future-Revised; LHA = Life History of Aggression questionnaire; HCR-20 = Historical Clinical Risk-20; SAPROF = Structured Assessment of Protective Factors for Riolence Risk; PPI-R = Psychopathic Personality Inventory-Revised; BIS-11 = Barrat Impulsiveness Scale-11; SAPS = Schedule for the Assessment of Psychotic Symptoms; CIRA = Cornell’s Instrumental-Reactive Aggression; HADS = Hospital Anxiety and Depression Scale; PSD = Posttraumatic Stress Disorder checklist; MCCB = MATRICS Consensus Cognitive Battery; BDHI = Buss-Durkee Hostility Inventory; STAS = Spielberger Trait Anger Scale; SES = Social Expression Scale; TRA = Theory of Reasoned Action

### Intervening variable

In this review, four studies examined moderating variables and five studies examined mediating variables. The variables and study outcome are as summarised in Table 5. All the studies focused on observing the role of the intervening factor in the relationship between risk of violence factors and aggression in forensic inpatient settings.

**Table 6:** *Significant Mediating Factors*

Study	Mediating Effect	Theme	Effect Size
[5]	Social cognition	Cognitive process	Cohen’s *d* = 0.46–1.82 (largest)
[64]	Moral cognition	Cognitive process	Eta2 = 0.17 to 0.62 (medium to large)
[66]	Cognitive component of the Theory of Reasoned Action (TRA)	Cognitive process	-

**Table 7:** *Non-Significant Effects*

Study	Moderating Effect	Mediating Effect	Effect size
[30]	Gender	-	-
[61]	Intellectual ability	-	OR = 0.49 (small to medium)
[62]	-	Self-deception	*r* = − 0.12 (small to medium)
[63]	Types of offence	-	OR = 1.01
[67]	Adverse childhood experience	-	*r* = 0.13–0.40 (small to medium)
[68]	-	Violent images	OR = 3.2–28.8

#### Mediating factors

Five studies reported on different mediating variables between risk of violence and aggression. A significant mediation effect was found in three studies (Table 6). The variables identified as significant mediators were social cognition (social reasoning task for managing emotion) [5], moral cognition [64], and the Theory of Reasoned Action (TRA) i.e. a person’s behaviour is determined by their intention to perform the behaviour and influenced by attitude and subjective norms [66]. In contrast, two studies that investigated self-deception, and violent visual as mediators yielded an insignificant outcomes [62, 68],] (Table 6).

#### Moderating factors

Four studies assessed the effects of moderating variables, namely intellectual ability, adverse childhood experience, gender, and offence type. However, they were all insignificant, indicating that none of the factors were associated with or influenced the direction and strength of the relationship between risk of violence and aggression [61, 63, 65, 67] (Table 7).

## Discussion

This paper aimed to conduct a scoping review of literature focusing to identify and explore intervening factors in the relationship between risk of violence and aggressive behaviour within the context of forensic psychiatric inpatients. The research trends in the topic area suggesting that the understanding of the relationship between risk of violence and aggression using intervening model is an emerging research area. Hence, it is not surprising our scoping suggest that the intervening variables has yet to be extensively explored. The evidence from the studies included remains insufficient to establish a conclusive outcome.

The insufficient data paired with other two significant reasons which are the methodological limitations and a lack of replicated studies. limits the discussion The methodological limitation was observed in some of the studies, such as small sample size, demographic heterogeneity, (e.g. gender, types of offences), and cross-sectional design. Pedersen et al. (2021) [70], mentioned that methodological challenges including issues on recruitment and representation may potentially affect the outcome quality of reviews studies. In this case, the forensic mental health population is relatively small but diverse, thus it is challenging to obtain a generally representative population [70]. Furthermore, poor sample representation of the population being studied may compromise the statistical significance of the results. The inherent nature of heterogeneity in forensic mental health services also affects the generalisability of study outcomes. One of the ways to overcome this is by ensuring transparency in reporting is recommended [70].

Our findings revealed that, research in the area of forensic inpatient suffers gender data gap. This finding corroborates the existing discussion pertains to gender influence on aggression that has been inconclusive. Among the plausible explanations is that secure facility population is predominantly male potentially due to higher rate of crime and criminal justice system involvement among males [71]. Some research supports the notion that gender influence in risk and aggression differs between males and females [72–74]. The differences were vastly discussed from the aspect of frequency, severity, and types of aggression. Huitema et al. (2021) [8], found female have higher aggression incidence with lower severity as compared to male inpatient and mostly engaged in autoaggression. Nature of aggressive incidents among the male inpatients in forensic facilities tend to be more severe.

However, despite these differences, gender in risk of violence assessment appeared trivial [75]. Making uniform conclusions about aggression in forensic facilities without gender consideration can lead to unsuitable prevention and management strategies. Last but not least, our scoping review did not capture the same variable used in two or more research. In other words, there are a lot of unexplored variables in the relationship being studied that warrant further research.

### Risk of violence

To the best of our knowledge, this is the first review that identify and describe the relationship between risk of violence and aggression via mediation-moderation model using the SPJ approach as a risk of violence assessment. The SPJ approach to risk of violence has revolve around moderation model and its relation to repeated aggressive behavior measured retrospectively within the forensic inpatients of gender imbalance. However, the insignificant outcome of the included studies, understudied mediation model using the SPJ or actuarial tool and the similar limitation of the studies involved warranted for further investigation.

In contrast, risk of violence measured using an independent risk factor that is highly associated to violence risk and aggressive behavior namely impulsivity, schizophrenic symptoms [64], neurocognitive impairment [5], and hostile personality trait [66] resulted in a significant mediation model. These factors are potent and embedded in the actuarial or SPJ assessment method such as the Historical Clinical Risk Management-20 (HCR-20) [75]. The HCR-20 subjectively assesses past and current aggression issues with specific attention among those diagnosed with mental illness, violent attitude, antisocial behaviour, as well as problems resulting from personality disorder or cognitive impairment [76].

In the context of estimating mediating and moderating effect in the connection between risk assessment and aggressive behavior among the forensic inpatients is less known. Despite the insignificant findings in the studies using risk of violence assessment tool, the lack of research in the focused area meant that it is not possible to reject the possibility of an intervening relationship between risk of violence and aggressive behaviour. This notion is supported as mediation study by Williams and Stansfield (2017) [77] using actuarial risk assessment in between the relationship of risk of intimate partner violence and recidivism produced a significant mediating effect. According to the research [77], applying a mitigating factor in between risk of violence and recidivism making application of violence risk tool as a suitable tool in the mediation model.

### Aggression

The finding highlights two methods were used to measure aggression as an outcome variable, namely incident observation (i.e., recorded from real-time observation, retrospective reporting and imagined response) and self-reported measures [5, 64, 66]. Physical aggression construct has been mainly used in this research area. Among the most plausible explanations of these findings is that physical aggression is a critical aspect to manage in secure environment as it poses risk to others safety [52, 78, 79]. Besides, physical aggression is more visible and measurable compared to other forms of aggression, making data collection easier. Moreover physical aggression are prioritized due to its significant legal and ethical implications [20, 80]. Consistent with the finding of a systematic review based on 74 studies of violence-measuring instruments for inpatient settings, we found that observable data, especially the overt aggression scales (OAS) variant, were more frequently used in the past ten years [81]. The study further classified the observable data as behaviour recording system that could be in the form of the OAS variant, daily recorded data, checklist, aggression forms, and severity index. This method offers valuable strength in the observation of triggers, motives, severity, victims, methods, and situational factors via a system of scoring and narratives.

On the other hand, Carr et al. (2019) [82] commented that the use of OAS could possibly induce errors in the interpretation of aggressive behaviours because of the disregard for the wide definitions of aggression in forensic mental health research [83]. This method is subjected to intra-rater bias, especially in events where data were collected in the past or by untrained personnel. This is common in the past ten years due to the increased application of violence risk assessment for aggression prediction purposes.

Furthermore, different facets of aggression from the perspective of patients can be obtained via self-reporting or interviews. Both methods can add value to the observation of aggression in research involving secure forensic facilities [84]. Understanding aggression from the patient’s perspective reveals individual experience, thoughts, behaviours, and emotions, all of which provide more in-depth information beyond mere incident reporting [7]. However, the self-reporting by patients must be interpreted with caution when assessing their thoughts, emotions, and behaviours as there is a tendency for patients to provide falsely positive impressions [85].

From the data we can see that only one study uses self-report measure of aggression that are driven by motivation (i.e., reactive or proactive). O’Reilly et al. (2019) [64] explained that reactive-proactive aggression can have different operating pathways. For example, people with schizophrenia spectrum disorder may portray less obvious signs of proactive aggression. On the other hand, cognitive impairment manifested by the schizophrenia diagnosis may be a determinant of reactive aggression [64]. Previous research supported the contrary in which higher motivation for aggression were strongly associated to increased impulsivity and psychopathy [84]. In other words, negative emotional states such as anger, hostility, rage, as well as jealousy between perpetrator and victim are expressed in the form of reactive or proactive aggressive behavior [86, 87]. Therefore, understanding aggression in an in-depth manner, whether it is reactive or proactive aggression, allows researchers to determine if the aggression is accompanied with emotional properties such as anger, rage, or hostility; or if it occurs in response to provocation or frustration as a strategy to reduce internal stress in relation to life limitations within an institution [88].

In keeping with this finding, a clearer understanding of the relationship to reactive-proactive aggression is necessary to develop targeted interventions and strategies to address and mitigate aggression among the forensic inpatients. According to Hornsveld & Kraaimaat (2022) [85] who study differences between self-reported and observed aggression among male forensic inpatients, concluded anger as a significant psychological factor to aggression and could present as state or trait characteristics. Thus they suggested the use of self-reported reactive and proactive aggression to gauge different perspectives on the underlying motivations and characteristics of aggressive behavior.

### Mediator’s role

From the review, three mediating factors were identified, i.e., moral cognition, social cognition, and cognitive TRA, all of which were shown to have a significant influence on aggressive behaviour among the forensic inpatients.

Moral cognition is defined as complex mental processes driven by the links of emotion, and cognitive process from moral judgement, decision-making, bias, and behaviour selection. These processes are later translated into attitudes, beliefs, and judgement, either resulting from correct or incorrect perceptions [89]. Delusions and hallucinations are a good illustration of moral cognition in which the impaired information processing, affecting what a person would consider as morally correct for a particular situation (moral cognition) [64]. Persons experiencing psychotic symptoms may frame a moral situation to fit to their moral appraisal and belief of a specific action. The reactive or proactive form of aggression would then be influenced by different moral cognitions, based on the types of psychotic experienced by the patients [64].

Additionally, emotional state may also influence the expression of violence, in which certain emotions may activate responses congruent to the cognition [90]. For example, loyal-betrayal moral cognition mediates persecutory delusion and reactive violence, which means that the delusion triggers an internal state of negative emotion such as anger, fear, or shame, subsequently activating the moral judgement that leads to reactive violence. As highlighted by Kamaluddin et al. (2017) [91], moral judgement can obscure the selection and application of moral judgement when executing certain behaviours. Similarly, in a study among the incarcerated juveniles, delinquent behaviour was observed more commonly among juveniles who lack maturity in moral judgement and those who reported more cognitive distortions [92]. The review finding was also supported by Hart and Ostrov (2020) [93] in which specific forms of aggressive behaviours were more closely related to moral cognition. This is because moral judgement can be distinctly conceptualised in the form as either reactive or proactive aggression in patients with psychiatric disorders.

The second mediating variable is the, social cognition, a sub-component of neurocognitive function. The neurocognitive function covers a wide range of functions from processing speed, verbal memory, and executive function to social cognition [94]. Therefore, the degree of impairment in relation to aggressive behaviour can be associated with the level of insight and functioning within the individual. The association between neurocognitive function and aggression is debatable due to complexity of aggression, measurement challenges, individual differences, or inconclusive causal direction [95]. A study among patient with schizophrenia found that impairment in executive functioning and social cognition are prominently associated to reactive aggression [96]. Notably, individual with cognitive impairment and history of violent offences involved more in impulsive aggression [97]. The inability to perceive and make sense of the surroundings would mediate an individual’s aggressive response style, often due to the pre-existing limitations in the neurocognitive function [5].

Despite the presence of risks such as neurocognitive limitation, a person would still be able to make social reasoning and judgement that subsequently affect the motivation of aggressive behaviour, depending on the person’s capacity and behavior consequences [98]. This finding is in concordance with empirical results that showed a person makes sense of their surroundings before they make a judgement in choosing direct or indirect forms of aggression as their response through social cognition [99]. This is further supported by a meta-analysis in which social cognition was established as an antecedent to criminal thinking that in return predispose to aggressive behaviour [100].

Various mechanisms of social cognition that are shaped by experience and personal preference can influence social reasoning within a specific situation before the selection of aggressive behaviour [101]. Certain situations are perceived as cues of social cognition before the individuals make an interpretation of the events and decide to react. In other words, studies that only observed impairment as larger cognitive functions could only be related to aggression through the total indirect effect of social cognition and risk of violence. For example, a person with executive impairment is prone to act aggressively due to his/ her inability to make social perceptions or inhibit impulsivity in any given risk status. Hence, neurocognitive impairment may play a more distal role in aggression while the identified mediators, i.e. social cognition play a more proximal role in aggressive incidents.

Lastly, the Theory of Reasoned Action (TRA) was also identified as a mediator in between risk of violence and aggressive behavior [66]. TRA includes three core components, i.e. intention, attitude, and subjective norm that can contribute to choices of action [102]. TRA believe cognition is centralised on the concept that individuals possess their own will to act based on their subjective norms, attitude, and intention. In any given situation, if a person had the intention to react aggressively, the intention is indeed a function of attitude towards aggression based on subjective norm. Hence, the TRA represent a chain of action, despite the debatable concept of distinguishing whether behaviour performed was a personal will or triggered by normative reasons [103, 104]. For instance, in a stressful situation, an individual may evaluate aggressive behaviour as an attitude condoning aggression as compared to in an unthreatened situation. The belief that the behaviour is appropriate within a subjective norm would then result in higher motivation, indirectly endorsing aggressive behaviour. Overall, there are very limited studies that evaluated TRA in an attempt to understand the mechanisms of social behaviour such as aggression [105, 106].

### Summary

This study set out to determine intervening factor between risk of violence and aggressive behavior. These findings suggest that in general, intervening factor may play role in the relationship between risk of violence and aggressive behavior among forensic inpatients. It was also shown that mediation process plays a significant role in the relationship. Interestingly, the identified mediation factors, i.e., moral cognition, social cognition, and cognitive TRA were all focused on the cognitive domain that highlighted the function of individual intrinsic factors.

The social cognition construct was developed based on the Social Learning Theory (SLT) whereby learning occurs in the social context [107]. Furthermore, the mediating factor through SLT emphasized that acquiring and maintaining behaviour is a dynamic and reciprocal interaction between persons, behaviour, and environment. Therefore, the social environment in which a person performs a specific or targeted behaviour offers important information about the mechanism of the behaviour.

The social cognition construct explains how experience predicts the occurrence of a behaviour, shapes behaviour engagement, and explains reasons for engagement through reinforcement, expectations, and expectancies [108, 109]. In this case, the social cognition concept can be readily applied to conceptualise aggression, as well as its prevention and management [110]. Six key constructs i.e., reciprocal, behavioural capabilities, observation learning, reinforcement, expectations, and self-efficacy are vital in the understanding of aggression. The principle of behaviour modification that is often used in the management of aggressive behaviour also applies certain elements of the social cognition model [111]. Moreover, this concept is more relevant to be applied among patient with mental illness and repeat offences [112].

Next, under the concept of human agency, the dynamic and reciprocal determinism reveals that a person can function as both an agent for change and a responder to change [113, 114]. Each function requires self-efficacy, i.e. the person’s confidence in his/her ability to take action and persistency appears to play a major role in influencing aggressive behaviour change, including the resistance towards resorting to aggression. Self-efficacy is also important in ensuring the effectiveness and maintenance of aggression management programme [115]. In this aspect, healthcare providers can make deliberate efforts to improve inpatients’ self-efficacy as an integral part in existing aggression and risk management strategies.

As abovementioned, moral cognition refers to the human capacity to experience and respond to situations of moral significance [116]. It has also been conceptualised as a set of capacities that allows people to engage with one another within acceptable social and moral norms. In other words, individuals must learn about norms, and make judgement about the norms, before making decision by considering the consequences of violating the norms [107]. The development process of moral cognition considers social cognition as the foundation body of learning morality [117]. Consistent with O’Reiley et al. (2019) [64], Hsu and Ouyang (2022) [118], impulsivity and personality features influence the moral reasoning of patients with schizophrenia who engage in repetitive aggression. Therefore, moral reasoning can come across as an agent of change for people with mental disorders via the strengthening of their willpower to engage in self-care skills, eventually improving interpersonal relationships and empowering decision-making for behaviour changes [118].

**Fig. 2:**
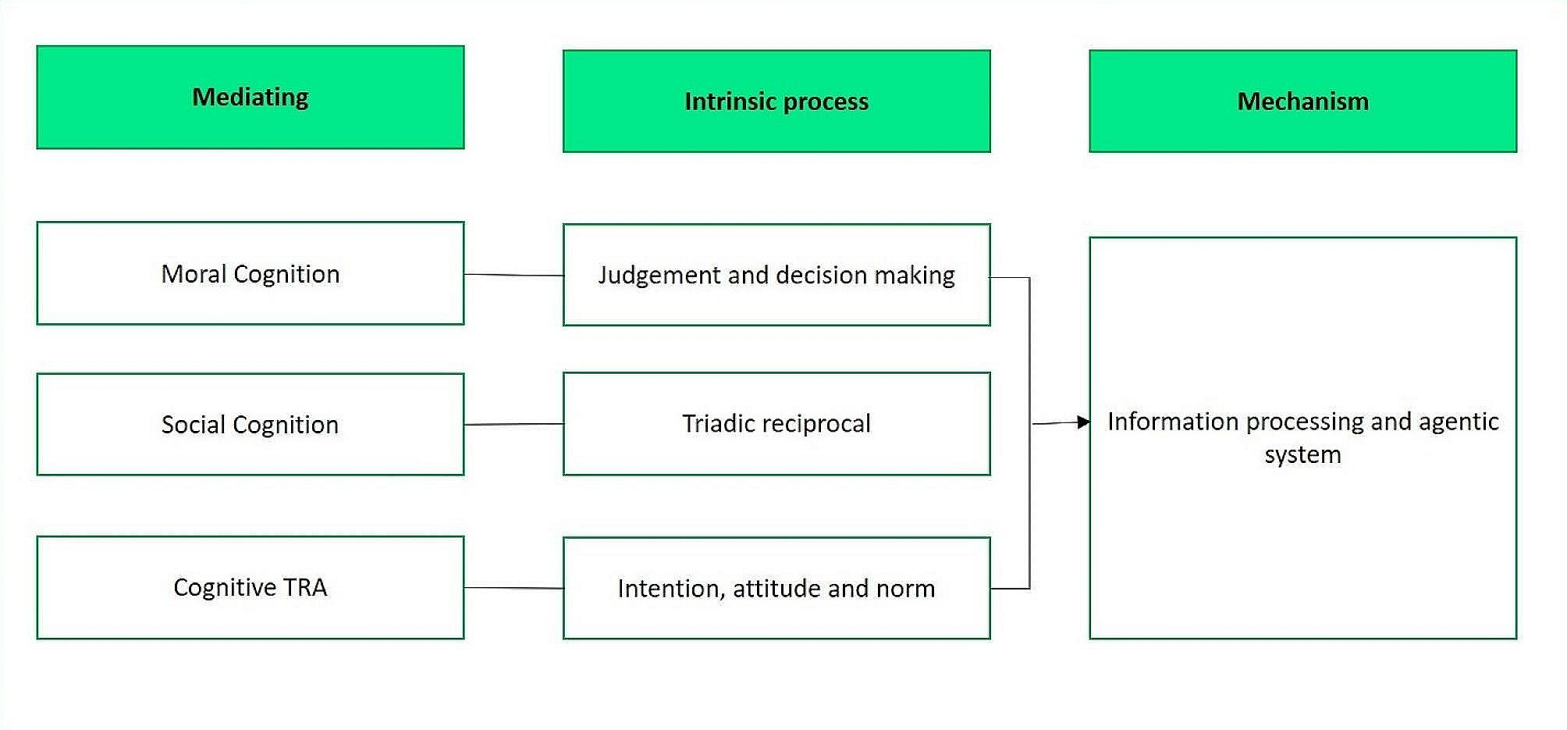
Mechanism of mediation

Generally speaking, cognition is an expression of human agency properties (intentions, forethoughts, self-reactiveness, and self-reflectiveness) that is central to the understanding of various behaviours, including aggressive behaviour. Thus, the mediation factor revealed in this review zoomed into personal cognition as an important factor in explaining the relationship between various facets of risk of violence and aggressive behaviour (Fig. 2). There are a few explanations for the relationship. Firstly, the mediation factors represent the proximate process between risk of violence and aggressive behaviour. Secondly, mediators may influence a person’s present internal state (physiological arousal, emotion, and cognition) prior to behavior. The interactions between these factors can increase the likelihood of aggressive behaviour or aggressive tendencies through agentic mechanisms (i.e., intention, forethoughts, self-reactiveness, or self-reflectiveness). This was highlighted as the role of human agency in Bandura’s Social Cognitive Theory [114]. Thirdly, the mediation factor can also influence the process of information processing, either via an unconscious automatic mode that involves very little cognitive effort or a reappraisal process that creates thoughtful action for affective purpose, goal, and intention. Figure 2 summarises the underlying process of the mediating factors.

These explanations can be further understood by delving into various theories and models such as the General Aggression Model (GAM), Information Processing Theory (IPT), Social Learning Theory (SLT), Theory of Reasoned Action (TRA), or General Personality and Cognitive-Social Learning (GP-CSL). GAM summarises various theories explaining aggressive behaviour by conceptualising aggression as an output to a chain of consequences that originated from the person or/and environment, followed by an inspection of the situation before deciding to respond [119]. Similarly, GP-CSL applies a perspective that identifies personally mediated control mechanisms, including but not limited to the attitude, values, and beliefs of an individual. The outcomes produce a powerful prediction of whether one is favouring aggression or resistance [35]. Next, IPT suggests that individuals with a history of aggressive behaviour often perceive and make a biased decision about social information, causing them to exhibit an increased tendency of aggression [120]. In short, the risk of violence within an individual can be a result of the reciprocal effects between cognition, the person as an agent, and the action of aggression [3, 119].

On an important note, we observe that significant results in the mediation studies share similar underlying mechanisms. For example, the interactional causal structure from each factor is rooted in the mechanism of information processing and belief system. Themes derived from the included studies highlighted cognition (i.e., information processing, knowledge structure, and belief system) to play a central role in the underlying mechanism of aggression. As such, human agency (person as an agent and object) explains the process of information processing, knowledge structure, and belief system automatically, supporting the theory that human agency plays a vital role in explaining the option of free will and volition behaviour [114]. More importantly, the cognitive process in human agency encapsulates humans as an agent and execution objects with the biggest potential to explain possible differences in risk of aggression and aggression responses. In other words, aggression is seen as an enactment of free will through cognitive structures that promote the choice of behaviour as the right or suitable course of action in the perceived social environment [1, 109, 121].

## Study limitations

There are certain limitations to this review. Firstly, there was a limitation in the scope of the literature search as only two academic databases were included that may led to the exclusion of some studies addressing the research question. Secondly, publications in only English language were included, thus excluding potentially relevant studies published in other languages. Thirdly, the small number of studies limited the analysis and conclusiveness of the findings, thus impacting the validity of the overall outcomes. The diversity of the studies included in this review means that the construct varies in different context. This makes comparison difficult and result should be treated with caution. Finally, the article included mostly cross-sectional, so conclusions about the influence of intervening factors on the relationship between risk of violence and aggressive behavior could not establish the causality pathway.

Nevertheless, in answer to the research question, intervening variable role in the relationship between risk of violence and aggressive behavior update the current understanding of the existing relationship. Furthermore, the exiting research suggest that the intervening variable could be further understood as human agency mechanism in between risk and aggressive behavior. However, future research is warranted.

## Conclusion

In summary, this review produces some level of evidence in supporting possible intervening factors in the relationship between risk of violence and aggression. Despite varying risk factors and violence measurements, there is an overall positive influence of moral cognition, cognitive intention, and social cognition on institutional aggression. However, considering the paucity of evidence, we cannot establish a clear conclusion pertaining to the intervening factors. Therefore, further studies are needed to investigate the aspect of human agency believed to be foundation of human ability as a mediator in the relationship. Furthermore, the limited number of studies investigating intervening factors also warrants more in-depth research. Nevertheless, our review added knowledge to the gap in the research area and in understanding complex phenomena of aggressive behaviours among forensic inpatients specifically to inclusion of intrinsic construct of aggression, proximal factors to aggression and strengthening risk of violence affect on aggression.

## Data Availability

The datasets used and/or analysed during the current study are available from the corresponding author on reasonable request.

## Abbreviations

WoS: Web of Science
PRISMA-ScR: Preferred Reporting Items for Systematic Reviews and Meta- Analyses-extension for scoping reviewa
PCC: Population/Concept/Concept
